# Immunometabolic Reprogramming by Black Soldier Fly (*Hermetia illucens*) Lipids in Monogastric Nutrition: From Receptor Crosstalk to the “Immune-Energy Sparing” Effect

**DOI:** 10.3390/ani16101501

**Published:** 2026-05-14

**Authors:** Ruxi Yuan, Xiaoyang Ma, Xiaochen Ma, Xiaoyi Jia, Hongbin Si

**Affiliations:** 1 Guangxi Key Laboratory of Animal Breeding, College of Animal Science and Technology, Disease Control and Prevention, Guangxi Grass Station, Guangxi University, Nanning 530004, China; xi-2008@163.com (R.Y.); 15006758505@163.com (X.M.); 19116884901@163.com (X.M.); 2 Guangxi Yankon Biotechnology Co., Ltd., Nanning 530004, China; 13607871066@163.com

**Keywords:** *Hermetia illucens*, medium-chain fatty acids, immunometabolic reprogramming, GPR84/PPARγ crosstalk, mitochondrial energy rescue, intestinal barrier

## Abstract

The transition to a post-antibiotic era necessitates novel interventions to mitigate gastrointestinal inflammation and optimize metabolic efficiency in monogastric animals. This review evaluates the *Hermetia illucens* (BSF) lipid matrix as an evolutionary signal sensor rather than merely a caloric substrate. The BSF lipid fingerprint—rich in lauric acid and bioactive co-factors—exerts a synergistic “entourage effect,” which is proposed to thermodynamically disrupt pathogenic membranes and engage GPR84/PPARγ crosstalk to silence sterile inflammation. Metabolically, medium-chain fatty acids bypass the CPT-1 bottleneck, enabling rapid mitochondrial ATP rescue that supports intestinal tight junction restoration. This targeted immunomodulation is hypothesized to underpin an “immune-energy sparing” effect—redirecting bioenergetic fluxes from inflammatory antagonism toward muscle protein deposition—a phenomenon that correlates with improved feed conversion ratios in vivo.

## Simple Summary

In nature, the ancestors of pigs and poultry frequently foraged for insects as a core dietary component. However, modern intensive farming systems rely heavily on plant-based fats like soybean oil, replacing the evolutionarily conserved insect lipid substrates. This dietary shift eliminates specific mucosal protective mechanisms in the gastrointestinal tract, creating a baseline for chronic low-grade inflammation in monogastric animals. This review explores the sustainable application of oil extracted from Black Soldier Fly (*Hermetia illucens*) larvae as a novel functional feed ingredient. We detail how this lipid matrix exerts dual core functions: directly disrupting the membranes of pathogenic microorganisms and modulating mucosal immune signaling, as hypothesized, via proposed GPR84/PPARγ dual-receptor crosstalk. The medium-chain fatty acids enriched in black soldier fly oil can alleviate local intestinal inflammation, while providing rapid energy substrates for the repair of the intestinal barrier. We further propose that integrating insect-derived lipids into feed may drive an ‘immune-energy sparing’ effect, potentially redirecting nutrients and energy from non-productive inflammatory responses toward muscle growth. This nutritional strategy has the potential to reduce diarrhea incidence and antibiotic use in livestock farming.

## 1. Introduction: The Evolutionary Recall Strategy

### 1.1. Evolutionary Tracing: Genetic Adaptation of Monogastric Ancestors’ Foraging Trajectories to Insect Medium-Chain Fatty Acid (MCFA) Signals

**Figure 1:**
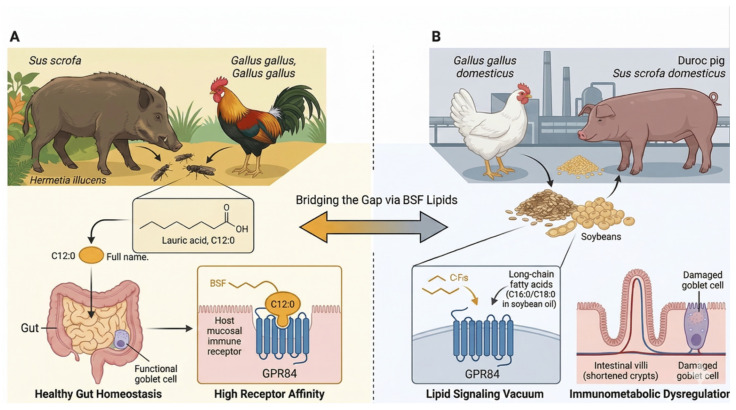
The evolutionary logic of black soldier fly (BSF) lipids in monogastric nutrition: from ancestral entomophagy to the modern “Lipid Signaling Vacuum.” (**A**): Under ancestral foraging conditions, regular insect consumption by *Sus scrofa* and *Gallus gallus* ancestors provided a consistent dietary supply of medium-chain fatty acids (MCFAs), primarily lauric acid (C12:0). This sustained input maintained high receptor affinity at the intestinal mucosal interface and supported healthy gut homeostasis, including intact functional goblet cells. (**B**): Modern intensive farming relies predominantly on seed-derived long-chain fatty acids (LCFAs, C16:0/C18:0 in soybean oil), which lack the MCFA ligands required to engage innate immune sensors such as GPR84 at the intestinal mucosa. This dietary shift creates a “Lipid Signaling Vacuum” and leads to a state of immunometabolic dysregulation. Center arrow: Reintroducing BSF-derived lipids into commercial feed is proposed to bridge this gap, restoring the evolutionarily conserved lipid-receptor signaling axis. Created with BioRender.com.

Monogastric species, specifically *Gallus gallus* (domestic chicken) and *Sus scrofa* (domestic pig), exhibit an evolutionary foraging trajectory where entomophagy constitutes a basal dietary behavior [1]. This ancestral dietary pattern drives a genetic and physiological adaptation to insect-derived biomolecules, particularly medium-chain fatty acids (MCFAs) such as lauric acid (C12:0), which are highly enriched in *Hermetia illucens* (black soldier fly, BSF) lipids [2,3]. Structurally and metabolically, MCFAs bypass bile salt emulsification and L-carnitine-dependent mitochondrial transport, are absorbed directly via the hepatic portal vein, and undergo rapid β-oxidation [2]. This metabolic pathway constitutes the core of the evolutionary recall mechanism, wherein the host gastrointestinal tract and innate immune sensors maintain conserved receptor affinities optimized for insect lipid matrices. Modern intensive husbandry predominantly uses seed-derived long-chain fatty acids (LCFAs), displacing ancestral lipid substrates and creating a structural Lipid Signaling Vacuum at the intestinal mucosal interface. Conventional plant oils used in intensive farming are dominated by C16-C18 long-chain fatty acids, with negligible MCFA content, failing to provide the conserved ligands for host innate immune sensors [2]. The absence of MCFA ligands disrupts homeostatic lipid-receptor signal transduction, driving intestinal villus deterioration, goblet cell depletion, and enteric dysregulation [2,3]. Reintroducing insect-derived MCFAs acts as a sustainable, climate-smart nutritional intervention [4] and we hypothesize it reactivates these dormant evolutionary signaling cascades, enabling the transition from passive caloric supply to active gastrointestinal immune reprogramming (Figure 1).

### 1.2. The “Lipid Signaling Vacuum” and Immunometabolic Dysregulation

Industrialized feed manufacturing standardizes macronutrient delivery but systematically depletes species-specific evolutionary lipid ligands, exacerbating the Lipid Signaling Vacuum at the mucosal interface. Conventional diets, defined by highly processed or oxidatively unstable lipid matrices, fail to supply essential MCFA substrates to mucosal innate immune receptors. Unbalanced or oxidized dietary lipids induce reactive oxygen species (ROS) accumulation, downregulate critical tight junction proteins (ZO-1, Occludin, Claudin-7α), and trigger intestinal epithelial damage [5]. Commercial feed formulations also act as vectors for environmental stressors, exerting baseline cytotoxic pressure on the gastrointestinal tract [6]. The combination of missing immunomodulatory ligands and persistent oxidative stress drives maladaptive hepatic lipid metabolism, upregulating pro-inflammatory cytokines including IL-1β and IL-8 [7], with this chronic low-grade inflammatory state defined herein as Immunometabolic Dysregulation. It is important to clarify that the concept of a ‘Lipid Signaling Vacuum’ refers specifically to the loss of evolutionarily conserved MCFA ligands and their unique metabolic and receptor-engagement properties, not to an absence of any lipid-mediated signaling. Long-chain fatty acids (LCFAs), including n-3 and n-6 polyunsaturated fatty acids, are established immunomodulators via pathways such as PPARα, GPR120, and TLR4. The ‘vacuum’ hypothesis posits that the displacement of ancestral MCFA inputs by a monoculture of plant-derived LCFAs creates a specific, unopposed pro-inflammatory bias, which the reintroduction of an MCFA-rich matrix is proposed to correct. Herein, we operationally define Immunometabolic Dysregulation in the gastrointestinal tract as a state characterized by at least two of the following: (i) a >2-fold upregulation of at least one pro-inflammatory cytokine in ileal or cecal tissue relative to a healthy foraging reference state; (ii) a significant reduction in transepithelial electrical resistance (TEER) or a decrease in the protein expression of tight junction components (ZO-1, Occludin) by ≥30%; and (iii) a shift in the gut metabolome indicating impaired oxidative phosphorylation, such as an increased lactate/pyruvate ratio or depleted ATP levels in mucosal biopsies. This definition provides a framework for future hypothesis-driven studies. Evidence shows that reintroducing BSF larvae into commercial diets restructures host tissue fatty acid profiles, selectively replenishing evolutionary lipid anchors like C12:0 and myristic (C14:0) acids [8,9]. We propose that this reintroduction of saturated medium-chain ligands terminates the signaling vacuum, neutralizes diet-induced oxidative stress, and redirects physiological flux from systemic inflammatory defense back to tissue accretion.

### 1.3. Defining BSF Oil: From Caloric Substrate to Evolutionary Signal Sensor

Evaluating *Hermetia illucens* (BSF) extract solely as a caloric substrate omits its primary functional capacity as an evolutionary signal sensor. We propose a working model in which, in the context of the host gastrointestinal tract, BSF oil may operate as an active evolutionary signal sensor. This functionality is driven by the entourage effect, wherein the primary lipid matrix serves as a biochemical delivery vehicle for a synergistic complex of MCFAs, endogenous antimicrobial peptides (AMPs), and structural polysaccharides (chitin/chitosan) [10,11]. It must be emphasized that the co-elution and functional concentration of these minor bioactive compounds (AMPs, chitin oligomers) are highly dependent on the extraction method. The ‘entourage effect’ as a fully integrated mechanism is best supported for minimally processed lipid fractions, whereas highly refined or solvent-extracted oils may lack many of the polar co-factors. This critical distinction is discussed in detail in Section 3.2 and Section 4.1. This unhindered metabolic trajectory is proposed to mediate a “mitochondrial energy rescue” effect within metabolically exhausted enterocytes, which may rapidly replenish the intracellular ATP pool allocated for the structural reconstitution of intestinal villi and tight junction barriers [2,3]. Simultaneously, the co-eluted polysaccharide fractions and AMPs exert targeted antimicrobial pressure, directly disrupting pathogen membrane viability while modulating host innate immune receptors to restructure the gut microbiome profile [12,13]. By integrating direct mitochondrial fuel provision with receptor-targeted immune stimulation, we hypothesize that the BSF lipid-polysaccharide-peptide complex may bridge the proposed “Lipid Signaling Vacuum”, and potentially re-establish the immunometabolic homeostasis that is systematically deactivated by conventional industrial diets [3,11].

### 1.4. The Aim of the Present Review

This review evaluates the “evolutionary signal sensor” hypothesis in monogastric production models, deconstructs the BSF lipid matrix from a caloric substrate to a targeted immunometabolic intervention, and translates evolutionary lipid adaptation theory into a quantitative formulation framework for precision veterinary interventions.

### 1.5. A Note on Terminology and Conceptual Framework

Throughout this review, we introduce several conceptual terms—including “evolutionary recall strategy,” “Lipid Signaling Vacuum,” “entourage effect,” and “mitochondrial energy rescue”—to organize and synthesize the available evidence into a coherent working model.

## 2. Methods

This article is structured as a narrative review. While no formal systematic review methodology (including PROSPERO pre-registration or dual independent risk-of-bias assessment) or meta-analysis was applied, the literature collation process was conducted with transparency and reproducibility in mind.

### 2.1. Literature Search Strategy

A systematic literature search was performed across three electronic databases: Web of Science (Core Collection), PubMed, and Scopus, covering the period from January 2003 up to March 2026. The following Boolean search string was applied to titles, abstracts, and keywords:

(“black soldier fly” OR “*Hermetia illucens*”) AND (“lauric acid” OR “MCFA” OR “medium-chain fatty acid” OR “lipid complex” OR “insect oil”) AND (“poultry” OR “swine” OR “pig” OR “broiler” OR “monogastric”) AND (“GPR84” OR “PPARγ” OR “tight junction” OR “intestinal barrier” OR “feed conversion” OR “growth performance” OR “microbiome” OR “immunometabolism”).

### 2.2. Study Selection and Data Synthesis

The search yielded a substantial body of peer-reviewed literature (numbering in the hundreds across databases). Due to the narrative scope of this review, articles were screened primarily based on relevance of the title and abstract to the core hypotheses (immunometabolic reprogramming, intestinal barrier function, and production performance in swine and poultry).

Given the conceptual focus on establishing a mechanistic framework, the synthesis prioritized:

- In vivo monogastric feeding trials reporting physiological endpoints.
- Mechanistic studies (in vitro, murine, or teleost models) providing direct evidence for the proposed GPR84/PPARγ crosstalk or mitochondrial energy rescue pathways.

Extracted data were synthesized via a narrative framework to establish causal links between BSF lipid biochemistry and macroscopic production outcomes. As this is a narrative review, a formal PRISMA flow diagram and quantitative bias assessment were not performed; limitations regarding species-specific evidence gaps are explicitly addressed in Section 5 (opening note) and Section 6.4.

## 3. Biochemical Architecture: The “Entourage Effect” of Insect Lipids

### 3.1. De Novo Synthesis of C12:0 and Its Biochemical Convergence with Host Mammalian Milk Lipid Structure

The BSF larval lipid profile does not passively mirror dietary substrates: larvae drive de novo synthesis of C12:0 and C14:0 acids using dietary carbohydrates as acetyl-CoA donors [14,15], with C12:0 preferentially esterified at the sn-2 position of the triacylglycerol backbone [14,16].

This specific spatial conformation is biochemically consistent with the lipid structure of mammalian colostrum and sow milk. During gastrointestinal transit, pancreatic lipase exhibits strict regiospecificity: it hydrolyzes fatty acids only at the sn-1 and sn-3 positions of the triacylglycerol backbone, leaving the sn-2 ester bond fully intact [14,15]. Sn-2-bound lauric acid from BSF oil thus escapes complete hydrolysis and is preserved as 2-monolaurin, which circumvents energy-intensive intracellular micellar reconstruction and undergoes direct passive diffusion across the enterocyte apical membrane to maximize lipid absorption kinetics [14]. By virtue of its sn-2 stereochemistry analogous to that of mammalian milk lipids—a trait particularly relevant to suckling piglets—de novo-synthesized BSF oil nonetheless transitions from a basic energy source to a structural requisite in both swine and poultry. This stereospecific absorption forms the structural basis for the entourage effect (the unhindered entry of C12:0 triggering downstream intracellular immune and metabolic cascades).

### 3.2. The “Entourage Effect”: Synergistic Defense Mechanisms of Lipid-Soluble Chitin, AMPs, and Insect Sterols Within the BSF Oil Matrix

The pharmacological efficacy of BSF extract extends beyond the stoichiometric concentration of lauric acid. Mechanical and specific solvent extraction protocols co-elute a bioactive matrix comprising amphipathic AMPs, lipid-soluble chitin/chitosan oligomers, and specific phytosterols alongside the primary triacylglycerol fraction [14,17,18]. This multi-component complex mediates the “entourage effect”, a sequential, multi-target defense mechanism that bypasses pathogen resistance pathways characteristic of single-molecule interventions.

The multi-component matrix drives sequential synergistic pathogen neutralization through three coordinated actions. First, co-extracted AMPs disrupt the bacterial outer membrane, facilitating unesterified C12:0 influx to induce irreversible pathogen inactivation [19,20,21,22]. Second, co-eluted chitosan residues further restrict microbial proliferation [21]. Third, embedded insect sterols integrate into host enterocyte lipid rafts, optimizing pattern recognition receptor clustering to calibrate localized immune transduction without systemic inflammation [14]. Through this matrix synergy, the BSF lipid complex operates as a coordinated biochemical defense system rather than an isolated energy substrate.

It is crucial to note that the empirical basis for this multi-component synergy is largely derived from studies using whole larval meal or mechanically pressed oil [17,19]. The extent to which co-eluted chitin and AMPs are present at physiologically effective concentrations in highly purified, solvent-extracted BSF oil is poorly characterized and likely negligible. Consequently, the ‘entourage effect’ should be considered a processing-dependent phenomenon, maximized in ‘minimally refined’ matrices, and this distinction must guide formulation strategies.

### 3.3. Molecular Editing Potential: Substrate-Driven Functional Lipid Fingerprinting and Matrix Superiority

The metabolic plasticity of BSF larvae enables the targeted manipulation of its fatty acid and sterol profile via dietary substrate modification, a process defined as molecular editing to generate a customized functional lipid fingerprint. Empirical evidence demonstrates that integrating specific organic side-streams—such as fermented agricultural by-products or marine macroalgae—into the larval rearing media directly remodels the triacylglycerol and phospholipid composition, optimizing the n-3/n-6 polyunsaturated fatty acid ratio and enriching the accumulation of specific phytosterols [23,24,25].

This substrate-driven plasticity establishes the Matrix Superiority of BSF oil over isolated, chemically synthesized medium-chain fatty acids or their derivatives, such as pure glycerol monolaurate (GML). In comparative physiological models, pure synthetic GML triggers significant cellular stress and apoptotic responses [26], while the intact BSF lipid matrix—delivering lauric acid in its natural stereospecific conformation alongside co-eluted sterols and bioactive peptides—avoids this lipotoxicity entirely. The delivery of the complete lipid fingerprint effectively downregulates lipogenic gene expression (FAS, ACC, SREBP-1) [27] and activates anabolic target-of-rapamycin (mTOR/s6k1) signaling without inducing cytotoxic stress or apoptotic cascades [26]. Consequently, natural BSF oil functions as an integrated regulatory complex rather than a singular chemical disruptor, establishing its inherent functional advantages in modern monogastric feed formulations.

## 4. Processing Engineering: The Physicochemical Determinant

### 4.1. Extraction Methodologies: Reconstruction of the “Functional Lipid Fingerprint”

The lipid extraction process operates as the Physicochemical Gatekeeper, dictating the final biochemical topography of the BSF extract. Extraction extends beyond volumetric physical separation; it chemically reconfigures the functional lipid fingerprint by modulating the co-elution of the triacylglycerol matrix with antioxidant chaperones, including carotenoids, tocopherols, and phenolics [28,29].

Conventional solvent extraction achieves high mass yields, while polar solvents improve antioxidant recovery, though residual solvent risks compromise feed safety [30,31]. Mechanical pressing provides a solvent-free methodology that preserves structural and immunomodulatory components. Temperature-controlled mechanical pressing preserves carotenoids that shield the lipid matrix against oxidative degradation [32]. Cold pressing configurations retain the emulsifying capacity of the residual protein meal and minimize structural hydrophobicity [31]. The interaction between upstream dehydration and downstream pressing strictly dictates terminal oxidative stability; freeze-drying coupled with mechanical pressing yields the lowest peroxide values during prolonged storage [33].

Supercritical CO_2_ extraction enables targeted preservation of the functional lipid fingerprint, recovering high lauric acid content and bioactive minor lipids with lower oxidation indices than solvent extracts [34]. Pre-extraction thermal processing acts as an initial structural control point: drying methods directly dictate FFA release, with freeze-drying inducing extensive lipolysis while microwave/oven drying minimizes FFA generation [35]. Consequently, formulating a functional BSF lipid additive requires exact calibration of these integrated extraction parameters to execute customized matrix reconstruction. We therefore conclude that the choice of extraction methodology is not only an engineering decision, but also a key determinant of the final immunometabolic potency of the BSF lipid matrix.

### 4.2. The Primary Oxidative Vulnerability: The Reverse Inhibitory Effect of POV and FFA on Biological Efficacy

The functional efficacy of the BSF lipid matrix is contingent upon maintaining oxidative homeostasis. Industrial processing vectors, including thermal exposure and dehydration methods, dictate the kinetic accumulation of free fatty acids (FFA) and primary oxidation products. Subjecting BSF biomass to non-thermal slaughtering (freezing) combined with freeze-drying triggers intense lipolysis, producing FFA levels as low as 21% [36]; however, under certain processing conditions, freeze-dried oils have been reported to reach FFA contents as high as 55.8–69.1% [35], illustrating the extreme variability that results from different processing parameters. Furthermore, high-temperature processing accelerates lipid peroxidation, increases oxidation product accumulation, and depletes the PUFA profile of BSF oil [37,38].

This disruption of oxidative stability constitutes the primary chemical limitation of BSF oil integration. Surpassing the peroxide value (POV) and FFA critical thresholds reverses the physiological trajectory from an immunomodulatory benefit to a cytotoxic stressor [39]. Although most current evidence derives from in vitro models, oxidized BSF oil has been shown to exhibit significant dose-dependent cytotoxicity, completely negating its anti-inflammatory benefits in intestinal epithelial cells [35,40,41]. While highly elevated FFA fractions exert localized antibacterial pressure against specific pathogens, their accumulation operates at the expense of overall lipid stability [36]. The systemic ingestion of oxidized lipids introduces reactive aldehydes into the gastrointestinal tract, compounding dietary oxidative stress [33,42]. For animal feed formulators, we propose a conservative provisional threshold of POV < 15 mEq O_2_/kg and FFA < 2%. While these figures are extrapolated from general feed-grade fat quality standards and are consistent with the low oxidation levels achieved under optimized BSFL processing conditions [33,37]. Dedicated dose–response studies are needed to validate this specific boundary for BSF lipids. Failure to control peroxide value (POV) and free fatty acids (FFA) transfigures the evolutionary signal sensor into an oxidative disruptor, negating its biological efficacy in monogastric livestock (broilers, turkeys, and weaned piglets).

### 4.3. Targeted Delivery Strategies: Microencapsulation for Site-Specific Release of Medium-Chain Fatty Acids in the Hindgut

Free MCFAs, predominantly C12:0 derived from BSF, exhibit rapid paracellular and transcellular absorption in the proximal gastrointestinal tract (stomach and duodenum) [43]. While this rapid uptake provides immediate hepatic energy, it prematurely depletes the active lipid matrix before it reaches the distal intestinal segments (ileum and cecum), which are the primary anatomical sites for pathogenic colonization and microbiota dysbiosis. Therefore, to maximize the immunometabolic reprogramming potential of the BSF lipid fingerprint, particularly the site-specific engagement of GPR84/PPARγ signaling in the distal intestine, we propose that advanced microencapsulation technologies serve as essential pharmacokinetic delivery barriers.

Polymeric encapsulation utilizing biopolymer blends—such as alginate-soy protein isolates or complex coacervates integrating high-amylose resistant starch—provides robust structural integrity against gastric acidic conditions and pepsin degradation [44,45]. These matrices restrict premature lipolysis and upper gastrointestinal absorption. Upon transit into the distal intestine, the pH-responsive swelling and specific enzymatic degradation of the encapsulant shell trigger a controlled, site-specific release of the lauric acid-rich core [44,45,46]. Furthermore, modifying the interfacial properties of the encapsulant materials through thermal treatment or constructing lipid-polymer hybrid nanoparticles can systematically alter the susceptibility of the lipid core to pancreatic lipases, finely tuning the release kinetics [47,48]. By shielding the active MCFAs until they reach the hindgut, microencapsulation maximizes the local concentration of antimicrobial lipids [44,45]. We propose that this targeted delivery strategy optimizes the physical interaction between C12:0 and pathogenic cellular membranes in the lower digestive tract, effectively translating dietary lipids into a localized intestinal defense mechanism without necessitating elevated dietary inclusion rates.

## 5. Molecular Logic: Reprogramming the Gut-Liver Axis

### 5.1. Critical Evidence Appraisal Note

Before detailing the proposed mechanisms, the reader is cautioned that the evidence for BSF lipid efficacy is heterogeneous and strongly context-dependent. As detailed in Section 6.4, null or negative outcomes are reported in specific circumstances, notably in high-biosecurity, low-stress environments or with oxidized lipid products. Therefore, the pathways described below represent a mechanistic framework primarily derived from optimal experimental conditions and specific model organisms, the generalizability of which requires extensive validation in target livestock species. A comprehensive analysis of outcome heterogeneity and conflicting findings is provided in Section 6.4.

It is important to explicitly note that most of the current mechanistic studies on the cellular and molecular effects of medium-chain fatty acids are conducted in murine, human, or teleost models. Direct in vivo validation of the proposed GPR84/PPARγ crosstalk, mitochondrial energy rescue effect, and downstream immunometabolic regulatory pathways in porcine and avian species remains limited. Significant species-specific differences exist between swine and poultry that directly alter the response to BSF lipid supplementation: (1) poultry have lower endogenous chitinase activity and faster gastrointestinal transit time, which differentially affects the bioavailability of BSF components—limiting lipid digestion while paradoxically mitigating chitin-induced viscosity; (2) broilers have higher hepatic de novo lipogenesis capacity, making them more responsive to the lipid-lowering effects of MCFAs; (3) weaned piglets have been reported to exhibit higher expression of fatty acid transporters in the jejunum, potentially contributing to a higher tolerance for MCFA supplementation. The mechanistic framework proposed in this review is extrapolated based on existing biochemical and physiological evidence, and species-specific differences in these pathways need to be confirmed through targeted experimental studies in monogastric livestock, as detailed in Section 6.3.

### 5.2. Physical Defense Layer: Thermodynamic Disruption of Phospholipid Bilayers by Lauric Acid at the Critical Micelle Concentration

Based on principles of membrane biophysics and evidence predominantly from in vitro studies, it is proposed that the antimicrobial and antiviral mechanisms of *Hermetia illucens*-derived C12:0 operate independently of genomic resistance networks, driving a structural disintegration governed by thermodynamic surfactant principles. As an amphiphilic molecule, C12:0 spontaneously partitions into the lipophilic core of pathogenic phospholipid bilayers. The rate-determining parameter for this membrane permeabilization is dictated by the electrostatic penalty of trans-bilayer flip-flop; intracellular leakage initiates at half the critical micelle concentration (CMC/2) and progresses to complete structural permeabilization as the concentration approaches the critical micelle concentration (CMC) [49].

Upon reaching the CMC threshold, the accumulation of C12:0 and its primary monoglyceride derivative (glycerol monolaurate, GML) alters the spontaneous curvature of the pathogenic phospholipid bilayer. This hydrophobic insertion triggers membrane instability and ultimate lysis [50], with micellar C12:0/GML achieving 100% rupture efficiency for high-curvature viral envelopes and bacterial membranes [51,52]. The physical perforation of the cell membrane triggers an immediate collapse of the transmembrane proton motive force (PMF), releasing low-molecular-weight proteins and inducing rapid cytolysis without the induction of compensatory mutational pathways [53]. This biophysical destabilization neutralizes the pathogen, suggesting the potential establishment of a primary, mutation-resistant physical defense layer within the host gastrointestinal tract.

### 5.3. Receptor Signaling Layer: GPR84-Mediated Immune Reprogramming

#### 5.3.1. The GPR84-MAPK/ERK Cascade and Acute Enhancement of Macrophage Phagocytosis

Pending direct validation in swine and poultry, the following model is extrapolated largely from murine and teleost data. Beyond thermodynamic membrane disruption, C12:0 functions as a specific endogenous ligand for G-protein-coupled receptor 84 (GPR84), a metabolic sensor constitutively expressed on macrophages and innate immune cells [54,55]. Notably, species-specific differences in this pathway require further validation in monogastric livestock, as detailed in the opening of this section. The binding of C12:0 to GPR84 triggers an intracellular conformational shift, initiating Gi/o-protein-coupled signal transduction [56,57,58], which competitively inhibits cAMP accumulation while rapidly inducing phosphorylation of the MAPK/ERK cascade [54,59].

The acute elevation of p-ERK and p-MAPK intracellularly reprograms the macrophage phenotype, shifting the cell from a resting state into a hyper-phagocytic state [58]. In vivo and in vitro validations confirm that GPR84 activation by medium-chain agonists directly amplifies bacterial adhesion and drives the physical engulfment of pathogens, multiplying the macrophage phagocytic index [58,60]. This Immune Reprogramming provides an immediate, acute defense mechanism upon pathogen exposure. These findings indicate that C12:0 enhances macrophage phagocytic activity via the GPR84-MAPK/ERK axis [58,60], unlike long-chain saturated fatty acids that trigger TLR4-mediated NF-κB activation and chronic inflammation [61]. C12:0-mediated GPR84 signaling does not appear to activate this maladaptive inflammatory cascade. We therefore hypothesize that dietary delivery of the BSF lipid matrix may function as a targeted immunological modulator, reinforcing mucosal pathogen clearance through receptor-specific phagocytic enhancement while avoiding the chronic sterile inflammation associated with TLR4 activation.

#### 5.3.2. Receptor Crosstalk: Dual-Sensor Modulation via PPARγ Activation and Transcriptional Repression of NF-κB

The immunomodulatory function of BSF lipids extends beyond membrane-bound GPR84 to a secondary transcription-level regulatory layer, and we hypothesize that these two pathways form synergistic dual-receptor crosstalk that underpins the balanced immune modulation of BSF lipids. Intracellular lauric acid acts as a selective partial agonist for the nuclear receptor PPARγ [62], exerting sustained anti-inflammatory effects via transcriptional regulation.

This intracellular activation of PPARγ is hypothesized to establish a critical biochemical crosstalk with the GPR84-mediated signaling cascade, forming a two-phase, synergistic immune regulatory loop. The core logic of this crosstalk is the spatial and temporal complementation of the two pathways, which enables BSF-derived MCFAs to simultaneously enhance pathogen clearance while limiting excessive tissue-damaging inflammation, with direct supporting evidence from existing mechanistic studies:

1. Temporal sequential synergy: The GPR84-mediated pathway is an acute, rapid membrane-initiated response that occurs within minutes of MCFA exposure. Activation of GPR84 by lauric acid rapidly enhances macrophage phagocytosis to clear invading pathogens, which is the first line of mucosal defense [58]. In contrast, the PPARγ-mediated pathway is a sustained, slow transcriptional regulatory response that occurs hours after MCFA internalization, which functions to resolve the inflammatory response after pathogen clearance, preventing the transition from acute protective inflammation to chronic sterile inflammation [63].
2. Spatial functional complementation: GPR84 is predominantly expressed on the surface of infiltrating macrophages and innate immune cells in the lamina propria, where it modulates the phagocytic phenotype of immune cells [60]. PPARγ is highly expressed in both intestinal epithelial cells and resident macrophages, where it not only suppresses pro-inflammatory cytokine transcription in immune cells, but also enhances tight junction integrity and barrier function in epithelial cells [64]. This spatial distribution enables BSF-derived MCFAs to simultaneously modulate both immune cells and epithelial barrier function via the two receptors.
3. Direct biochemical intersection via ERK-mediated PPARγ modulation: Downstream effectors of GPR84 activation provide a direct biochemical link between the two pathways. GPR84 ligation by lauric acid induces rapid phosphorylation of ERK via the MAPK cascade [58]. Activated ERK, in turn, contributes to the modulation of PPARγ transcriptional activity, forming a positive regulatory loop that coordinates the two arms of the immune response [65,66].
4. Downstream signaling convergence via NF-κB: The two pathways converge on the NF-κB signaling cascade to form a closed functional loop. GPR84 activation enhances the phagocytic capacity of macrophages without triggering TLR4-mediated NF-κB overactivation [61], while activated PPARγ directly sequesters NF-κB components in the cytosol, blocking its nuclear translocation and transcription of pro-inflammatory cytokines (TNF-α, IL-6) [65]. This dual regulation ensures that the mucosal immune system maintains sufficient pathogen clearance capacity, while avoiding the tissue damage and metabolic cost of chronic NF-κB activation.

Critically, the evidence for this crosstalk model is derived almost entirely from murine and teleost models. Direct in vivo validation of GPR84 activation, GPR84–MAPK/ERK-mediated phagocytosis, PPARγ partial agonism, and PPARγ–NF-κB crosstalk by C12:0 has not been performed in poultry or swine. In poultry, only indirect gene expression data exist for PPARγ–NF-κB signaling [27], with no direct pathway validation. In swine, none of the five mechanistic nodes have been experimentally investigated.

**Figure 2:**
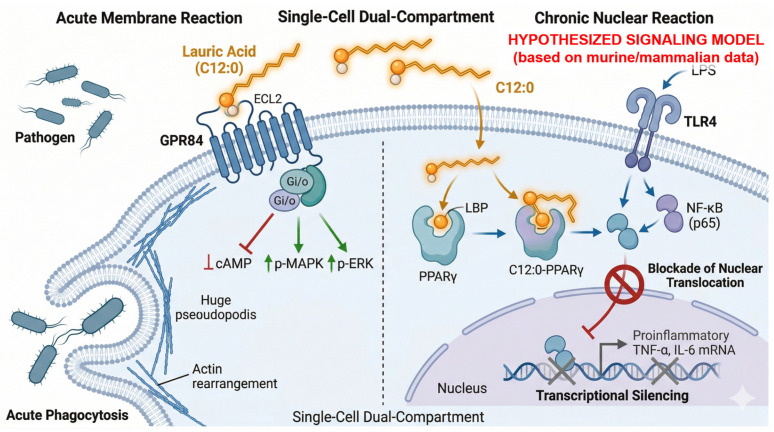
Hypothesized dual-receptor immunometabolic crosstalk mediated by *Hermetia illucens*-derived C12:0 in macrophages. This model is based primarily on murine and mammalian data and has not been directly validated in swine or poultry. The lipid matrix executes a spatial dual-sensor modulation. (**Left**) At the plasma membrane, extracellular C12:0 specifically binds to the G-protein-coupled receptor 84 (GPR84), triggering a Gi/o-dependent cascade that inhibits cAMP while acutely phosphorylating the MAPK/ERK pathway. This is proposed to rapidly shift the macrophage into a hyper-phagocytic state for pathogen clearance. (**Right**) Concurrently, intracellularly translocated C12:0 acts as an endogenous partial agonist for the nuclear receptor PPARγ. The ligand-bound PPARγ complex is proposed to physically sequester the NF-κB (p65) subunit within the cytosol, effectively blocking its nuclear translocation and executing the transcriptional silencing of sterile pro-inflammatory cytokines. Green arrows (↑) indicate activation/phosphorylation; red lines (⊥) denote inhibition or physical blockade. GPR84, G-protein-coupled receptor 84; PPARγ, peroxisome proliferator-activated receptor gamma; NF-κB, nuclear factor kappa B; MAPK, mitogen-activated protein kinase; ERK, extracellular signal-regulated kinase; cAMP, cyclic adenosine monophosphate. Created with BioRender.com.

Through this spatial and temporal dual-receptor crosstalk, these findings suggest that the BSF lipid matrix may effectively reprogram the localized immune response, arresting tissue-damaging sterile inflammation in the intestinal mucosa without compromising the baseline phagocytic capacity required for pathogen clearance [67] (Figure 2).

### 5.4. Energy Metabolism Layer: Mitochondrial Energy Rescue

#### 5.4.1. Accelerated Bioenergetic Flux: Bypassing the Damaged FATP4 and CPT-1 Rate-Limiting Systems

In mammalian and teleost models, it has been shown that during states of acute inflammation or metabolic stress, the conventional bioenergetic reliance on LCFAs is severely compromised. The intracellular transport and mitochondrial oxidation of LCFAs depend on a cascade of rate-limiting membrane translocases—specifically Fatty Acid Translocase (FAT/CD36) and Fatty Acid Transport Proteins (FATPs)—and the Carnitine Palmitoyltransferase (CPT-1 and CPT-2) shuttle system [68,69]. Inflammatory stimuli and oxidative stress downregulate these enzymatic complexes, precipitating the cytosolic accumulation of unoxidized LCFAs [70]. This bottleneck induces lipotoxicity, mitochondrial dysfunction, and intracellular ATP pool depletion [71,72].

Based on their biochemical properties, *Hermetia illucens*-derived MCFAs, predominantly C12:0 and octanoic (C8:0) acids, are thought to circumvent these damaged transport networks, driving a stoichiometric “energy rescue”. Due to their specific carbon chain lengths and favorable physicochemical thermodynamics, MCFAs do not require CD36/FATP-mediated membrane translocation. More critically, within hepatic and intestinal epithelial tissues, MCFAs cross the outer and inner mitochondrial membranes independently of the CPT-1/carnitine shuttle [73,74]. Metabolic flux analyses utilizing etomoxir—a specific irreversible inhibitor of CPT-1—confirm that MCFA β-oxidation and subsequent ATP generation proceed unabated even when LCFA mitochondrial entry is pharmacologically or pathologically blocked [73,75,76].

This carnitine-independent trajectory establishes an accelerated bioenergetic substrate flux. Upon entering the mitochondrial matrix, MCFAs are directly activated into acyl-CoAs by medium-chain acyl-CoA synthetases, providing an immediate and continuous flux of acetyl-CoA into the tricarboxylic acid (TCA) cycle [74,77,78]. This mechanism rapidly replenishes exhausted intracellular ATP reserves required for maintaining enterocyte tight-junction integrity and sustaining macrophage phagocytic activity. By bypassing the LCFA-induced lipotoxic cascades and overcoming the CPT-1 metabolic bottleneck, the BSF lipid matrix is proposed to support uninterrupted cellular bioenergetics and facilitate tissue survival during acute immunometabolic crises.

#### 5.4.2. Bioenergetic Basis for Tight Junction (ZO-1/Occludin) and Cytoskeletal Reconstruction

The structural integrity of the intestinal epithelial barrier is governed by mitochondrial oxidative phosphorylation (OXPHOS) and intracellular ATP availability. The assembly, maintenance, and membrane localization of tight junction (TJ) complexes—specifically zonula occludens-1 (ZO-1), occludin, and claudins—coupled with their anchorage to the apical F-actin cytoskeleton, constitute a highly energy-dependent physical process [79,80]. Under conditions of oxidative stress or lipotoxicity induced by long-chain saturated fatty acids, enterocytes experience mitochondrial uncoupling, leading to a critical depletion of the intracellular ATP pool [81]. This bioenergetic deficit prevents TJ assembly, resulting in the rapid internalization and cytoplasmic redistribution of transmembrane proteins and the fragmentation of the F-actin cytoskeleton [79,80]. These structural collapses quantitatively manifest as a decrease in transepithelial electrical resistance (TEER) and a concomitant increase in paracellular macromolecular flux [79,82].

The continuous ATP generation provided by BSF-derived MCFA β-oxidation is hypothesized to act as the fundamental bioenergetic substrate for intestinal barrier restoration in monogastric animals. By bypassing mitochondrial transport bottlenecks, C12:0 maintains stable, high-efficiency ATP production without inducing the OXPHOS uncoupling or ROS accumulation characteristic of LCFA overload [81]. This accelerated bioenergetic flux sustains intracellular energy homeostasis and facilitates the activation of adenosine monophosphate-activated protein kinase (AMPK) [82,83]. AMPK activation functions as an absolute prerequisite for the accelerated reassembly of tight junctions; it directly drives the plasma membrane relocalization of ZO-1 and its physical cross-linking with the reconstituted actin network [83]. Notably, species-specific differences in this pathway require further validation in monogastric livestock, as detailed in the opening of this section. Consequently, the sustained bioenergetic flux derived from BSF-derived MCFA metabolism may structurally reverse epithelial hyperpermeability, which supports our hypothesis that the mechanical barrier defense of the gut-liver axis is fundamentally subordinate to, and driven by, mitochondrial metabolic efficiency.

### 5.5. Cross-Organ Closed Loop: Regulatory Effects of Gut-Derived Lauric Acid on Hepatic Steatosis and Acute-Phase Proteins

The immunometabolic functionality of BSF lipids extends beyond the localized intestinal mucosa to execute a systemic cross-organ closed loop via the gut-liver axis. Anatomically, unlike LCFAs that necessitate micellar packaging into chylomicrons for lymphatic transport, gut-derived MCFAs, predominantly lauric acid, bypass the lymphatic system. They are absorbed directly into the portal vein, establishing a rapid, high-concentration substrate flux directly to the hepatic sinusoids.

Upon reaching the liver, this MCFA influx remodels hepatic lipid metabolism. In vivo pathological evaluations demonstrate that full-fat BSF biomass, which retains the intact lipid matrix, may help prevent hepatic steatosis [84]. Whether this hepatoprotective effect is recapitulated in poultry and swine remains to be directly investigated. Conversely, the administration of defatted BSF diets induces hepatic lipid accumulation and elevates serum alanine transaminase (ALT) levels [84]. Most of these pathological evaluations are conducted in aquaculture species and murine models. The long-term effects of BSF lipid supplementation on hepatic lipid metabolism and liver health in pigs and poultry, especially in breeding animals with high metabolic load, require further longitudinal investigation. The continuous β-oxidation of portal MCFAs competitively inhibits de novo lipogenesis pathways, leading to a linear reduction in circulating triglycerides, total cholesterol, and hepatic oxidative stress markers, such as malondialdehyde (MDA) and nitrotyrosine [85,86].

Concurrently, this metabolic reprogramming at the hepatocyte level modulates the activation threshold of resident Kupffer cells. The attenuation of hepatic lipotoxicity directly suppresses the transcription and systemic release of acute-phase proteins (APPs). Clinical veterinary models confirm that optimal dietary inclusion of BSF lipids linearly decreases the plasma concentration of C-reactive protein (CRP), a primary APP, without compromising basal liver or renal functionality [85,87]. This targeted downregulation of CRP signifies the termination of the chronic inflammatory feedback loop between the intestinal barrier and the liver. Ultimately, we propose that the gut-derived BSF lipid matrix may act as a systemic metabolic modulator, translating localized mucosal defense into global hepatic homeostasis and alleviating systemic immunometabolic dysregulation in monogastric animals.

## 6. Phenotypic Synthesis and Formulation Matrix

### 6.1. Morphological Enhancement and Targeted Remodeling of the L/E Ratio

The molecular cascades and bioenergetic rescue mechanisms proposed in this review are supported by phenotypic outcomes reported in existing in vivo studies, which are collated and synthesized herein, specifically manifesting within the intestinal histomorphology and the microbial ecological niche of monogastric animals.

In vivo morphological evaluations demonstrate that dietary inclusion of BSF larvae meal at tested levels (1% to 9%) can enhance intestinal absorptive surface area, primarily through increased villus height and villus height-to-crypt depth ratio in the duodenum and jejunum [88,89]. Histomorphometric datasets confirm a quadratic increase in villus height and villus height-to-crypt depth (VH/CD) ratio in the intestine of monogastric models [89,90]. This macroscopic elongation of the villi is the direct physical manifestation of the sustained mitochondrial ATP flux, which supports continuous epithelial cell proliferation and the structural reinforcement of tight junctions without inducing inflammatory tissue remodeling.

Concurrently, the intrinsic antimicrobial dynamics of the BSF lipid fraction induce a targeted compositional shift in the hindgut microbiome. 16S rRNA amplicon sequencing data across both poultry and swine models confirm a systemic competitive exclusion mechanism [90,91]. The inclusion of BSF lipids significantly attenuates the relative abundance of opportunistic pathogenic taxa within the Proteobacteria phylum, driving a marked reduction in Enterobacteriaceae populations, specifically *Escherichia-Shigella* and *E. coli* [91,92]. Simultaneously, the microenvironment enriches commensal populations, driving the proliferation of *Lactobacillus* spp. and *Bifidobacterium* spp. [91,93]. This diametric population shift fundamentally maximizes the *Lactobacillus*/Enterobacteriaceae (L/E) ratio. In industrial formulation matrices, an elevated L/E ratio serves as a primary, quantifiable biological marker for gut homeostasis, confirming that the BSF lipid matrix effectively mediates microecological remodeling and positions it as a highly efficacious substitute for conventional antibiotic growth promoters.

### 6.2. Quantitative Assessment of the “Immune-Energy Sparing” Effect: Flux Redirection from Inflammatory Antagonism to Protein Deposition

Chronic low-grade gastrointestinal inflammation is well-documented to redirect amino acids and bioenergetic substrates from somatic growth toward the systemic acute-phase response in monogastric production, creating a futile “metabolic tax” that impairs feed efficiency [94]. The dietary integration of the BSF lipid matrix is hypothesized to systematically neutralize this metabolic tax via the proposed “immune-energy sparing” effect, the mechanistic framework of which is delineated in this review.

Existing in vivo quantitative models provide correlative evidence for this hypothesis: optimal BSF supplementation has been shown to directly downregulate mucosal pro-inflammatory transcripts (IL-6, IL-18) while amplifying anti-inflammatory signals (IL-13) in broilers and weaned piglets [27]. This localized mucosal immune quenching translates into a systemic “immune-sparing” phenotype. Hematological profiling across multiple monogastric trials demonstrates that BSF lipids drive a linear reduction in basal inflammatory tone. For instance, one trial reported a reduction in circulating white blood cells and total lymphocytes of 35.9% and 47.7%, respectively, at the highest inclusion level, alongside a 4-fold reduction in CD3+ T-lymphocytes [95]. This targeted suppression of non-productive immune activation may uncouple the classic “immune-metabolic trade-off” in intensive livestock production, unlocking a substantial pool of conserved bioenergetic substrates and amino acids [96].

The conserved nitrogen and ATP fluxes are hypothesized to be redirected toward terminal skeletal muscle protein deposition, which underpins the improved growth performance observed in multiple in vivo trials. At the transcriptional level, BSF lipids have been shown to actively reprogram nutrient partitioning in reduced-protein dietary matrices: they downregulate primary lipogenic genes (FAS, ACC) to reduce non-functional abdominal fat pad accumulation [27], while activating anabolic mTOR/s6k1 signaling in skeletal muscle [26]. These molecular changes correspond to statistically significant increases in hot eviscerated carcass weights and crude protein content in breast and thigh tissues of broilers, as reported in multiple feeding trials [97,98].

For industrial feed formulators, the most commercially relevant phenotypic endpoint of this metabolic reprogramming is the Feed Conversion Ratio (FCR). For instance, a controlled trial by Vilela et al. (2021) [95] reported a 10% improvement in FCR in broilers fed increasing levels of full-fat BSF meal, as one data point in the literature. It is critical to clarify that this FCR improvement cannot be exclusively attributed to the proposed “immune-energy sparing” effect, and multiple confounding factors must be considered. These include (1) differences in gross energy and digestible energy content between BSF lipids and conventional plant oils; (2) enhanced nutrient digestibility driven by improved intestinal barrier integrity and gut microbiota remodeling; (3) optimized amino acid utilization from the concomitant protein fraction of full-fat BSF meal; (4) differences in basal diet formulation and animal health status across trials.

The improved nutrient digestibility driven by enhanced intestinal barrier function may directly increase amino acid and energy availability for growth, while the higher digestible energy content of BSF oil relative to some plant oils may also contribute to improved FCR. That said, the weight of existing correlative evidence supports the proposed “immune-energy sparing” effect as a core mechanistic driver of the observed production benefits. This hypothesis warrants further causal validation via targeted in vivo trials. By alleviating chronic sterile inflammation and rerouting metabolic fluxes toward skeletal muscle accretion, the BSF lipid matrix may transition from a simple caloric feed ingredient to an active metabolic partitioning agent, with the potential to improve economic returns in precision commercial monogastric production.

Future studies employing pair-fed experimental designs, iso-energetic and iso-nitrogenous diet formulations, and targeted knockout models of GPR84/PPARγ signaling are required to definitively validate the quantitative contribution of the “immune-energy sparing” mechanism relative to other established benefits of BSF lipids.

### 6.3. Species-Specific Thresholds and Proposed Starting Points

The successful translation of the BSF lipid matrix from experimental cellular models to commercial-scale formulations mandates the abandonment of generic inclusion paradigms. Poultry and swine have four core physiological differences that directly determine the optimal inclusion threshold of BSF lipids, which form the biological basis for the species-specific dose recommendations in Table 1:

1. Gastrointestinal anatomy and transit time: Poultry have a significantly shorter gastrointestinal tract and faster digesta transit time (2–4 h in broilers, versus 24–36 h in weaned piglets) [99]. This rapid transit minimizes the anti-nutritional viscosity effects of chitin, allowing broilers to tolerate higher full-fat BSF meal inclusion (up to 12%) than weaned piglets (up to 10%) [98,100]. The longer retention time in piglets, while improving chitin digestion, increases the risk of intestinal viscosity and dysbiosis at high inclusion levels.
2. Endogenous enzyme activity differences: Poultry have extremely low endogenous chitinase activity throughout the gastrointestinal tract, making them highly susceptible to the anti-nutritional effects of excessive chitin from full-fat BSF meal [93,99]. In contrast, weaned piglets have measurable gastric and pancreatic chitinase activity [4], which partially mitigates the anti-nutritional effects of chitin at moderate inclusion levels; however, this endogenous chitinase capacity is limited and can be overwhelmed at higher dietary inclusion levels, leading to growth depression [99].
3. Lipid metabolism differences: Fast-growing broilers have a high capacity for de novo hepatic lipogenesis, making them highly responsive to the lipogenic gene-suppressing effects of BSF-derived lauric acid, with a lower optimal oil inclusion level (1–3%) to avoid hepatic lipid overload [27,101]. Weaned piglets have lower hepatic lipogenesis, and require a higher oil inclusion level (2–5%) to achieve sufficient anti-inflammatory and barrier-repair effects.
4. Physiological stress patterns differ: Broilers experience chronic, low-grade metabolic stress throughout the 42-day production cycle, requiring sustained, low-dose immunomodulation. Weaned piglets face acute, transient intestinal barrier damage and pathogenic challenge during the 2–4-week post-weaning period, requiring higher, short-term doses of BSF lipids to repair the intestinal barrier and prevent diarrhea.

Exceeding these optimal physiological thresholds saturates endogenous digestive capacities, triggering hepatic steatosis and intestinal dysbiosis, which entirely negate the aforementioned “immune-energy sparing” benefits. To prevent the lipid matrix from traversing from an immunomodulator to a cytotoxic oxidative stressor, formulators must enforce an absolute universal oxidative safety threshold of POV < 15 mEq O_2_/kg across all species. Clinical veterinary trials have explored distinct inclusion ranges and reported varied immunological and performance outcomes for each biological system. These are organized in Table 1 as proposed, research-informed starting points for future validation. The ranges presented should be viewed as suggestions based on existing trials, not as industrial guidelines.

**Table 1:** Proposed research-informed starting points for BSF lipid intervention in monogastrics.

Target Model & Physiological Stressor	Tested & Proposed Inclusion Range ^1^	Oxidative Safety Boundary (POV)	Micro-Level Targeted Signatures (Receptor/Mucosal)	Reported Phenotypic Outcomes	Biological Threshold Constraint (Overdose Risk)
Avian: Fast-Growing Broilers (Metabolic load & chronic sterile inflammation)	BSF larvae oil: 1–2% [101]; Partially defatted BSF larvae meal: 5–10% (negative effects at 15%) [102]; Full-fat BSFL meal: 12.5% and 25% SBM replacement [98]	<15 mEq O_2_/kg	↓ Lipogenic transcription (FAS, ACC) [27].	~10% FCR improvement in one trial [95]; ↑ Eviscerated carcass & breast muscle accretion in some trials [95,97]; Competitive exclusion of cecal Enterobacteriaceae [98]; No ARG enrichment at low BSFLM inclusion [98].	Dietary chitin overload inducing upper-GI viscosity and early feed intake depression.
Avian: Turkeys & Waterfowl (Hepatic lipogenesis & oxidative susceptibility)	Defatted BSF larvae meal: 5% (turkeys) [103]; Partially defatted BSF larvae meal: 3–9% (Muscovy ducks) [85]	<15 mEq O_2_/kg	↓ Hepatic oxidative stress (MDA, Nitrotyrosine) in ducks fed up to 9% defatted BSF meal [85].	No signs of hepatic steatosis in ducks fed up to 9% defatted BSF meal [85]; Terminal meat quality attributes unaffected in reported trials [85,103].	Exceeding optimal thresholds disrupts baseline PUFA deposition profiles.
Swine: Weaned Piglets (Post-weaning tight junction collapse & ETEC challenge)	BSFL oil: 2–6% [104]; Whole dried BSF prepupae: 4–8%.	<15 mEq O_2_/kg	Accelerated ZO-1/Occludin & Claudin-3 reconstitution in ETEC-challenged piglets [105].	↑ Anti-inflammatory cytokine IL-10 [105]; ↓ ETEC-induced weaning diarrhea in challenge trials [105]; Improved ADG in piglets fed whole BSF prepupae	Proximal intestine chitinase saturation triggering dysbiosis [99].

^1^ Note: These ranges represent levels tested in published trials and are proposed only as starting points for future validation. They should not be interpreted as validated commercial optima, as formal multi-dose response studies are still lacking for most categories. POV < 15 mEq O_2_/kg is a proposed provisional safety threshold extrapolated from general feed-grade fat quality standards. Upward and downward arrows represent increase and decrease, respectively.

### 6.4. Critical Appraisal of the Evidence: Heterogeneity and Conflicting Findings

While the immunometabolic benefits of BSF lipids are supported by a growing body of literature, a critical, unbiased appraisal of the evidence reveals significant heterogeneity in reported outcomes, alongside several studies reporting no significant beneficial effects or even negative impacts of BSF lipid/meal supplementation.

#### 6.4.1. Conflicting and Negative Outcomes in Published Studies

Several well-designed in vivo trials have reported no significant beneficial effects of BSF lipid supplementation in monogastric animals, and these null findings are fully consistent with the core sources of heterogeneity identified in this review. For example, a study in broilers found that BSF larvae fat supplementation did not alter cecal microbiota composition, intestinal barrier function, or growth performance compared to conventional soybean oil, with no significant improvement in FCR observed [92]. This null result can be directly attributed to two key factors: first, the trial was conducted in a high-biosecurity, specific-pathogen-free (SPF) environment with low pathogenic challenge, which eliminates the inflammatory background required for the immunomodulatory effects of BSF lipids to manifest; second, the basal diet was a balanced, high-nutrient-density commercial formula, which limits the “Lipid Signaling Vacuum” that BSF lipids are proposed to resolve.

Furthermore, excessive dietary inclusion of BSF products—whether as full-fat meal or as extracted oil—is associated with distinct negative effects, although the underlying mechanisms differ by product type. In broilers, full-fat BSF meal inclusion exceeding 15% significantly reduced feed intake and growth performance, driven by chitin-induced increases in digesta viscosity in the upper gastrointestinal tract [99]. Similarly, in Xuefeng black-bone chickens, 5% BSF larvae meal reduced jejunal VH and VH/CD ratio, accompanied by an increase in the relative abundance of potentially pathogenic genera such as Staphylococcus [88]. In weaned piglets, BSF oil inclusion exceeding 8% induced intestinal dysbiosis and reduced nutrient digestibility, attributable to saturation of endogenous lipase activity [93,104].

#### 6.4.2. Core Sources of Outcome Heterogeneity

The inconsistent outcomes across studies are primarily driven by five modifiable factors, which are critical for standardizing the application of BSF lipids in monogastric feed:

1. Oxidative quality of BSF oil: As detailed in Section 4.2, the peroxide value (POV) and free fatty acid (FFA) content of BSF oil directly determine its biological efficacy. Oxidized BSF oil acts as a cytotoxic stressor, rather than an immunomodulatory additive, which is the primary source of negative results in many trials [37].
2. Dietary inclusion level and basal diet composition: The beneficial effects of BSF products (oil or meal) generally follow a quadratic dose–response relationship, with both insufficient and excessive inclusion leading to non-significant or negative outcomes. Additionally, benefits are more pronounced in low-protein, high-stress diets, while minimal effects are observed in balanced, high-nutrient-density commercial diets [27].
3. Animal physiological stage and health status: Most positive studies are conducted in weaned piglets or fast-growing broilers, which are under high metabolic stress and intestinal barrier damage risk. In healthy adult animals or grow-finish pigs with stable immune function, the immunomodulatory benefits of BSF lipids are significantly diminished [90,105]. Notably, the disruption of intestinal barrier integrity in weaned piglets can lead to a cascade of systemic effects beyond localized gut dysfunction. Increased intestinal permeability facilitates the translocation of bacterial lipopolysaccharides (LPS) and other microbial components into the portal circulation, triggering systemic inflammatory responses that can manifest as swine inflammation and necrosis syndrome (SINS), a condition linked to gut-derived microbial translocation and hepatic inflammation [106]. Furthermore, this systemic inflammatory state induces sickness behavior, characterized by reduced feed intake, lethargy, and decreased social interaction, which further compounds the post-weaning growth check [107]. These systemic consequences highlight the importance of early nutritional interventions, such as BSF lipid supplementation, aimed at restoring intestinal barrier function and mitigating the progression from localized gut inflammation to systemic immunometabolic dysregulation.
4. BSF oil processing and composition: Extraction method, drying process, and larval rearing substrate directly alter the lauric acid content, co-eluted bioactive components, and oxidative stability of BSF oil [32,35]. For BSF meal, the degree of defatting determines the residual lipid content and the ratio of protein to chitin, both of which influence digestibility and intestinal health outcomes.
5. Trial environment and pathogenic challenge: The antimicrobial and anti-inflammatory effects of BSF lipids are amplified in high-pathogen-challenge, poor hygiene environments, while minimal benefits are observed in high-biosecurity, clean production systems with low environmental pathogenic pressure [90,92,93].

This critical appraisal confirms that the beneficial effects of BSF lipids are not universal, but are highly dependent on product quality, inclusion level, animal model, and trial conditions [27,32,37,90,92]. Standardization of these parameters is essential for reproducible application in commercial production.

A formal, quantitative quality appraisal across all cited trials—including a systematic classification of oil oxidative status, biosecurity level, and dietary formulation—is beyond the scope of this narrative review. Such an appraisal, ideally incorporating a standardized scoring system for study quality, constitutes an essential next step for a future systematic review and meta-analysis. Qualitatively, the majority of positive in vivo studies reviewed here were conducted in commercial or semi-commercial settings with moderate pathogenic challenge, where the risk of using highly oxidized oil is relatively low. However, this qualitative assessment requires systematic verification, and the possibility that publication bias favors positive findings under these conditions cannot be excluded.

### 6.5. Safety Considerations and Regulatory Hurdles for Commercial Deployment

The translation of BSF lipids from experimental feed additive to a globally traded commodity ingredient is contingent upon addressing legitimate safety, quality, and regulatory concerns [108], which are systematically delineated below.

#### 6.5.1. Core Safety Risks and Control Thresholds

1. Heavy metal bioaccumulation risk: *Hermetia illucens* larvae can bioaccumulate heavy metals, particularly cadmium, lead, and arsenic, when reared on contaminated organic substrates. While the lipid fraction of BSF larvae has a significantly lower heavy metal accumulation level compared to the protein fraction, strict substrate selection and mandatory heavy metal screening of the final lipid product are non-negotiable prerequisites for feed safety. Maximum residue limits must align with the regulatory standards for feed ingredients in the target market [108].
2. Mycotoxin and microbial contamination risk: Mycotoxins present in larval rearing substrates can be partially transferred to the larval biomass, while minimally processed BSF oil may carry pathogenic microorganisms such as *Salmonella* spp. and *Clostridium perfringens*. Validated thermal processing steps and strict microbial testing are mandatory to eliminate these risks, with thresholds aligned with national and international feed safety regulations.
3. Oxidative safety risk: As detailed in Section 4.2, oxidized BSF oil with elevated POV and FFA content induces intestinal oxidative stress, cytotoxicity, and reduced growth performance in monogastric animals. A proposed provisional safety threshold of POV < 15 mEq O_2_/kg and FFA < 2% should be adopted pending dedicated dose–response validation for all commercial BSF lipid products, to ensure biological efficacy and avoid adverse effects.
4. Allergenicity risk: Residual insect-derived proteins in BSF oil may induce potential allergenicity, and refined extraction processes that remove residual protein fractions can effectively mitigate this risk.

#### 6.5.2. Global Regulatory Status

The regulatory approval status of BSF-derived ingredients varies significantly across global markets. In the European Union, BSF oil has been approved for use in all animal feed since 2021, while in the United States, BSF larvae meal and oil are generally recognized as safe (GRAS) for use in poultry and swine feed. In China, BSF larvae oil has been included in the Catalogue of Feed Raw Materials (2013), with clear usage specifications for monogastric animals. However, inconsistent regulatory standards across markets create barriers to global trade, highlighting the urgent need for international harmonization of BSF lipid quality and safety standards.

The safety thresholds outlined in this section directly inform the four pillars of the proposed “Global Feed Insect Lipid Physicochemical Grading Standard” (Section 7.1), which aims to harmonize quality and safety requirements for BSF lipids across global markets.

## 7. Conclusions and Future Trajectories

### 7.1. Resolving Batch-to-Batch Variability: The Imperative for a “Global Feed Insect Lipid Physicochemical Grading Standard”

The establishment of a ‘Global Feed Insect Lipid Physicochemical Grading Standard’ is a core prerequisite for the standardized commercial application of BSF lipids. This proposed framework is intended to complement and harmonize with existing quality schemes and standards widely adopted in the feed industry, and for each pillar, established laboratory methods are already available. We propose a stratified classification matrix anchored by four quantitative pillars, directly derived from the mechanistic frameworks and safety thresholds established in this review:

Pillar I: Pharmacological Lipid Density (The Effector Matrix): Establishing minimum standardization thresholds for C12:0 enrichment and essential co-eluted bioactives. Consistent stoichiometric thresholds are mathematically required to guarantee the thermodynamic disruption of pathogenic phospholipid bilayers at the CMC, and to support the proposed GPR84/PPARγ dual-receptor crosstalk without triggering systemic inflammation.

Pillar II: Oxidative Homeostasis Boundary (The Bioenergetic Constraint): Proposing a conservative, provisional safety ceiling for primary oxidation products (POV < 15 mEq O_2_/kg) and hydrolytic degradation parameters (FFA < 2%). The POV threshold is extrapolated from general feed-grade fat quality specifications, for which standardized iodometric or potentiometric determination methods are well established [37,38]. Strict oxidative auditing should be implemented to prevent lipid peroxidation, with the understanding that dedicated dose–response studies are needed to validate this specific boundary for BSF lipids.

Pillar III: Forced Safety Thresholds (The Non-Negotiable Prerequisite): Establishing mandatory maximum residue limits for heavy metals (cadmium, lead, arsenic), mycotoxins (aflatoxin B1, deoxynivalenol), and total viable bacterial count (including *Salmonella* spp. and *Clostridium perfringens*), aligned with the EU, US, and Chinese regulatory standards for feed ingredients. This pillar is the non-negotiable prerequisite for all graded products, to eliminate food safety risks in the livestock production chain.

Pillar IV: Structural Matrix Integrity (The Pharmacokinetic Delivery): Quantifying the ratio of intact triacylglycerols to free fatty acids. This ratio accurately predicts upper-gastrointestinal absorption kinetics and dictates the architectural necessity for advanced microencapsulation strategies to ensure the site-specific release of MCFAs in the pathogen-rich hindgut.

### 7.2. Macro Vision: Operationalizing the “One Health” Paradigm in the Post-Antibiotic Era

The impending realities of the post-antibiotic era require a fundamental paradigm shift in intensive animal husbandry—transitioning from prophylactic pharmacological dependence to precision immunometabolic regulation. The mechanistic framework delineated in this review positions the BSF lipid matrix not merely as a functional feedstuff, but as a promising biological technology for sustainable livestock production. By simultaneously mediating the thermodynamic lysis of pathogenic membranes and the proposed GPR84/PPARγ-mediated dual-receptor modulation of sterile inflammation, BSF lipids provide a promising, commercially scalable alternative to in-feed antibiotic growth promoters. This targeted nutritional intervention offers a nutrition-based strategy to reduce reliance on in-feed antibiotics, and may thereby contribute to mitigating the agricultural propagation of antimicrobial resistance (AMR) genes.

Beyond the organismal level, the commercial deployment of BSF lipids intrinsically operationalizes the “One Health” triad—the inextricable optimization and synergy of Animal, Human, and Environmental health. Through the trophic upcycling of organic side-streams into high-value lipids, the BSF-mediated nutrient upcycling system offers a conceptual pathway for decoupling monogastric protein production from the ecological pressures associated with soybean cultivation and the depletion of pelagic fish stocks, although quantitative life-cycle assessments specific to BSF oil in monogastric diets remain limited [109]. Furthermore, the documented ‘immune-energy sparing’ effect drives a macroscopic redirection of bioenergetic fluxes, transferring metabolic currency from futile inflammatory antagonism toward terminal protein deposition, thereby improving overall feed efficiency.

Ultimately, the integration of *Hermetia illucens* lipids transcends the conventional boundaries of feed formulation to become a foundational ecological technology. Integrated into a closed-loop circular bioeconomy, precision immunometabolic optimization in livestock transitions from a localized formulation strategy to a systemic intervention, contributing to the mitigation of agricultural AMR transmission and optimizing global feed conversion efficiency.

### 7.3. Limitations of the Current Evidence Framework

Before translating the proposed immunometabolic framework into practice, several fundamental limitations of the current evidence base must be explicitly acknowledged.

1. Mechanistic Validation Deficit

This review’s central mechanistic thesis—the proposed GPR84/PPARγ dual-receptor crosstalk, the mitochondrial energy rescue pathway, and their convergence on the “immune-energy sparing” effect—rests on pathways that have not been directly observed or experimentally validated in swine or poultry. As reviewed in Section 5.3.2, the receptor-level evidence is derived almost entirely from murine, human, and teleost models. Extrapolation of these mechanisms to monogastric livestock relies on the assumption that the relevant receptors (GPR84, PPARγ) and downstream signaling components are functionally conserved across species. While this assumption is plausible given the evolutionary conservation of these pathways, it remains untested in the target species. All molecular diagrams presented in this review (Figure 2) should therefore be viewed as roadmaps for essential future research rather than as established in vivo facts.

2. Narrative Methodological Constraints

This article is structured as a narrative review. Although the literature search was conducted with transparency in mind (see Section 2), no formal systematic review protocol was applied: there was no PROSPERO pre-registration, no dual independent screening of titles and abstracts, and no formal risk-of-bias assessment of individual studies. Consequently, the literature selection, while systematic in intent, is susceptible to selection bias. The conclusions drawn in this review should be interpreted with this methodological context, and the synthesis presented here is intended to generate hypotheses for future systematic investigation rather than to provide definitive answers.

3. Potential Publication Bias

The narrative synthesis presented in this review may be further skewed by a file-drawer effect, whereby studies reporting null, negligible, or negative effects of BSF lipid supplementation are less likely to be published, or are published in journals with lower visibility and indexing coverage. The heterogeneous outcomes discussed in Section 6.4—including several well-designed trials reporting no significant improvement in performance or immune markers—are therefore likely an underestimation of the true prevalence of null findings. A formal assessment of publication bias, through funnel-plot analysis or equivalent methods, would require a systematic review and meta-analysis, which is beyond the scope of the current work.

4. Absence of Quantitative Synthesis

The decision to not perform a meta-analysis limits the generalizability of our conclusions. The overall effect size, its confidence interval, and its heterogeneity across studies have not been statistically estimated. As a result, the species-specific inclusion ranges presented in Table 1 should be interpreted as qualitative summaries of tested levels, not as statistically derived optima. A quantitative synthesis, ideally stratified by species, production stage, and BSF product type, is an essential next step.

### 7.4. Operational and Biological Limitations

While the immunometabolic efficacy of the BSF lipid matrix is robustly supported by cellular and short-term in vivo models, several operational and biological limitations must be acknowledged. First, the technological transition from crude mechanical pressing to advanced SC-CO_2_ extraction and targeted microencapsulation introduces substantial capital expenditure (CapEx). However, we posit that the proposed “immune-energy sparing” effect will offset these initial processing premiums in high-density production systems. This effect is correlated with up to a 10% improvement in feed conversion ratios reported in in vivo studies and the complete elimination of sub-therapeutic antibiotic costs.

Second, the regulatory compliance and commercial registration of BSF lipids are continually challenged by substrate-induced compositional fluctuations. Ensuring labelling consistency necessitates the stringent standardization of larval rearing substrates prior to lipid extraction. Finally, current phenotypic data collation studies are predominantly restricted to single production cycles. There remains a critical deficit in longitudinal, multi-generational toxicological tracking. Future investigations must prioritize multi-omics frameworks to evaluate the lifelong impact of continuous BSF lipid administration on hepatic enzymatic load, renal clearance capacity, and transgenerational microbiome stability. Addressing these gaps will definitively secure the status of BSF lipids as a foundational pillar in veterinary nutrition.

### 7.5. Research Priorities

Crucially, several of the core hypotheses advanced in this review await direct experimental validation. The “evolutionary recall” hypothesis, for instance, could be tested through comparative genomic analyses examining the evolutionary conservation of MCFA-sensing receptors across insectivorous and herbivorous lineages. Receptor binding assays and co-crystallization studies could further quantify the affinity of insect-derived lipids for these receptors relative to plant-derived LCFAs. The proposed “mitochondrial energy rescue” mechanism could be directly interrogated using CPT-1 inhibition models in enterocyte cell lines, measuring ATP flux and tight junction reassembly kinetics in the presence of purified BSF lipids versus isolated MCFAs. Systematic testing of these hypotheses will be essential to transition the conceptual framework presented here from a compelling theoretical model to a mechanistically validated paradigm.

To transition the field forward, we propose the following prioritized research agenda, directly addressing the key limitations identified in this review:

Priority 1 (Causal Validation of Core Hypothesis): Directly test the “immune-energy sparing” hypothesis using pair-fed, iso-energetic, iso-nitrogenous diets in weaned piglet and broiler models of low-grade inflammation, with precise quantification of whole-body energy partitioning via indirect calorimetry and tissue-specific metabolic flux analysis.

Priority 2 (Receptor Crosstalk Validation): Employ CRISPR-Cas9-mediated knockout of GPR84 and PPARγ in porcine and avian intestinal organoids and primary macrophages to determine whether the anti-inflammatory and barrier-protective effects of BSF-derived lauric acid are strictly dependent on these two receptors, and to validate the proposed direct crosstalk mechanism.

Priority 3 (Species-Specific Dose–Response Standardization): Conduct a systematic, head-to-head comparative study across broilers, turkeys, and weaned piglets using a standardized BSF oil preparation, to isolate the influence of gastrointestinal anatomy, endogenous enzyme activity, and receptor expression on dose–response relationships, hindgut microbiome modulation, and growth performance.

Priority 4 (Longitudinal Safety Assessment): Implement a multi-generational feeding trial in broiler breeders and swine sows, to assess the cumulative effects of lifetime BSF lipid consumption on reproductive performance, progeny gut health, hepatic and renal function, and potential transgenerational epigenetic programming.

### 7.6. Conclusions

In conclusion, we hypothesize that the BSF lipid matrix may function as a potent immunometabolic modulator in monogastric animals, capable of driving an “immune-energy sparing” effect. However, it is crucial to acknowledge that this framework is primarily supported by mechanistic extrapolation from non-target species and correlative in vivo data. Direct experimental validation of the GPR84/PPARγ crosstalk, mitochondrial energy rescue, and their quantitative contribution to the “immune-energy sparing” effect in swine and poultry is urgently needed before definitive commercial recommendations can be made.

## Data Availability

No new data were created or analyzed in this study.
